# High-performance surface plasmon resonance fiber sensor based on cylindrical vector modes

**DOI:** 10.1038/s41598-023-31524-9

**Published:** 2023-03-20

**Authors:** Vahid Sharif, Hassan Pakarzadeh

**Affiliations:** Department of Physics, Shiraz University of Technology, Shiraz, Iran

**Keywords:** Biophysics, Optics and photonics

## Abstract

Cylindrical vector modes with azimuthal polarization and low transmission loss are proposed for the first time to be utilized in a novel design of a surface plasmon resonance (SPR) sensor based on a circular photonic crystal fiber (C-PCF). A C-PCF with a ring of air holes in the cladding is designed where a gold layer with a thickness of 44 nm is coated on the outer cladding surface. The optimal geometric parameters are determined using the finite-element method (FEM) for a high-quality TE_01_ mode and high sensitivity of the sensor. The proposed SPR sensor shows high sensitivity for analyte refractive index (RI) ranging from n_a_ = 1.29 to 1.34 over the wavelength range of 1400–2000 nm. It is expected that the proposed sensor can sense low concentrations of hemoglobin, lymphocytes and monocytes of red and white blood cells which are effective in diagnosing the progress of cancer tumors. The maximum sensitivity of 13,800 nm/RIU is obtained in the refractive index environment of 1.33–1.34. The sensor resolution is of the order of 10^−6^ and the amplitude sensitivity reaches its maximum of 2380 RIU^−1^ at n_a_ = 1.30 which is the highest value ever reported. Our proposed sensor shows high sensitivity and simultaneously simple design with high performance.

## Introduction

In recent years, higher-order modes in optical fibers have shown significant applications in different areas of photonics such as optical communications^1^. Although higher-order modes in conventional fibers are usually formed as linearly polarized (LP) modes, new designs of optical fibers such as circular photonic crystal fibers (C-PCF)^2–4^, have been able to generate the orbital angular momentum (OAM) and cylindrical vector modes that can be propagated orthogonally along the fiber^5^. In the C-PCFs, OAM modes are generated from the linear combination of even and odd modes in the same order while TE_01_ and TM_01_ are the only pair modes that are independently propagated without combination and hence are the so-called cylindrical vector modes^6^. The use of TE_01_ in the sensing performance of C-PCF-based sensors can be introduced as a new idea because TE_01_ has significant optical properties such as high intensity, symmetric propagation, low confinement loss and azimuthal polarization.

The absorption of electromagnetic waves in a structure consisting of dielectric and metal is introduced as surface plasmon resonance (SPR) technique, which is highly influenced by the structure shape and dielectric/metal permittivity^7^. In recent years, this technique has been introduced and examined to achieve optical sensors in diverse structures such as prisms, waveguides, and fibers^8–11^. The performance of SPR sensors reaches from the excitation of free electrons on the interface of the plasmonic metals (such as Au, Ag, Cu, etc.) and dielectrics so that the frequency of incident light and the frequency of electrons are matched and hence the surface plasmon wave (SPW) is generated^10^. Due to the extreme sensitivity of the SPR on the refractive index variations of a surrounding medium, SPR sensors have been able to show a diverse range of applications in chemistry, environmental monitoring, medical diagnostics, biochemical, etc.^12,13^.

For the first time, the configuration of a fiber-optic SPR sensor was introduced by R. C. Jorgenson in 1993 to measure the refractive index of chemical solutions^7^. Compared to prism-based SPR sensors that require mechanical arms to launch light at specific angles, fiber-based SPR sensors are very compact with more freedom in design where the metallic layer can be coated anywhere depending on the sensor configuration. Also, fiber-based SPR sensors offer remote sensing capabilities for required applications ^14^. So far, many fiber-based SPR sensors have been designed and proposed that are completely different in terms of geometry, the array of air holes in Photonic Crystal Fibers (PCFs), materials and dimensions, amongst them the most common design is the D-shaped sensor^14–17^.

The fundamental mechanism of all fiber-based SPR sensors is the coupling between the core mode and the surface plasmon polariton (SPP) mode under phase-matching conditions^13^. Amongst the PCF-based SPR sensors, those formed with either internal metallic-coated^18,19^ or nanowires^20,21^ usually encounter problems due to infiltrating the sample/analyte liquid into the air holes of the photonic crystal structure and have more fabrication difficulties and less flexibility. Also, they suffer high confinement loss if the fiber core is surrounded by the metallic layer^22^. Contrarily, in external sensing approaches^23,24^ and D-shaped sensors where the metallic layer is coated on the external surface and the sensor is floated in a sample/analyte flow channel, transmission loss is reduced and they are easier to fabricate and use. Although various designs of fiber-based SPR sensors have been proposed, there are few practical efforts with experimental results in which simpler configurations have been reported. One is a hexagonal D-shaped SPR sensor that has reported a wavelength sensitivity of 21,700 nm/RIU from the simulation results which agreed well with the experimental results^25^. In a similar structure, the influence of the polish depth on the sensitivity of a D-shaped SPR sensor has been investigated experimentally and the highest sensitivity was reported 7381 nm/RIU in the sensing range of 1.40–1.42^26^. In another experimental effort, but with a different structure of photonic crystal fiber, the impact of adding a graphene layer on increasing the sensitivity has been examined and the maximum sensitivity of 2290 nm/RIU has been obtained^27^. Meanwhile, theoretical and simulation works have been able to report much higher sensitivities and theoretically respond to different refractive index environments due to fewer limitations in designing and adding plasmonic layers. One is a unique design of the SPR sensors configured with a multi-core flat fiber (MCFF) and numerically demonstrated a high sensitivity of 23,000 nm/RIU in the refractive index environment of 1.47–1.475^10^. Adding graphene layers on the metal layer^16,23,28^ or a combination of different sensing layers^19,29^ in fiber-based SPR sensors have been recent struggles to increase the sensitivity of the sensor so that in a numerical work, the hybrid sensing layer of Au/chalcogenide in a D-shaped sensor has increased the sensitivity of the sensor up 17,600 nm/RIU^30^. Also, tuning the refractive index sensitivity of a D-shaped sensor has been shown numerically by changing the chemical potential of graphene coated on the gold surface^31^. However, amplitude detection versus those spectral-based is a more practical approach for evaluating sensor performance because the measurements can be performed at a single wavelength and the challenges and costs of spectral manipulation are eliminated^32^. The maximum amplitude sensitivity ever reported is 1086 RIU^−1^, which has been obtained numerically in a highly sensitive D-shaped SPR sensor^14^, while our simulation results show an improvement factor better than two.

PCF-based D-shaped sensors are usually designed with more complex cladding to achieve a strong birefringence since only one state of polarization (*x* or *y*) is desired. Contrarily, the birefringence properties for radially (TM_01_) and azimuthally (TE_01_) polarized modes appear strongly in all OAM-supported fibers^33^, including C-PCFs, so is not required to reconfigure the fiber to realize the sensor. This can be considered as an important advantage, especially in coupling the sensor and fiber. Therefore, only by reducing the rings of cladding air-holes in C-PCFs and coating a metallic layer on the external surface, the azimuthal TE_01_ mode can influence well the metallic layer, excite the free electrons and then generate the SPP mode. Since the C-PCF core does not change, despite the strong coupling between the TE_01_ and SPP modes, the transmission loss remains low. Therefore, C-PCF-based SPR sensors can be introduced as a new generation of high-performance sensors with a simpler structure.

In this study, to the best of our knowledge, we propose for the first time a highly sensitive SPR sensor based on the cylindrical vector modes in the C-PCF. The sensor ring core is formed by considering the central air hole and a ring of air holes in the cladding on which a gold layer is coated. The proposed sensor is introduced with the most optimized geometric parameters using a large number of simulations based on the finite-element method (FEM). The advantages of TE_01_ mode in the C-PCF-based SPR sensor are discussed and it is shown that TE_01_ mode has enough intensity with simultaneously non-degenerate behavior and low confinement loss. Also, strong coupling between TE_01_ and SPP modes is shown and the sensor performance is assessed via calculation of the wavelength and amplitude sensitivities (${S}_{\lambda }$ and ${S}_{A}$), resolution (R) and figure of merit (FOM). In addition, the effect of gold layer thickness on the performance of the proposed sensor is discussed. Finally, the obtained results are compared with those of high-sensitive sensors reported in the literature.

### Sensor design and theoretical framework

**Figure 1:**
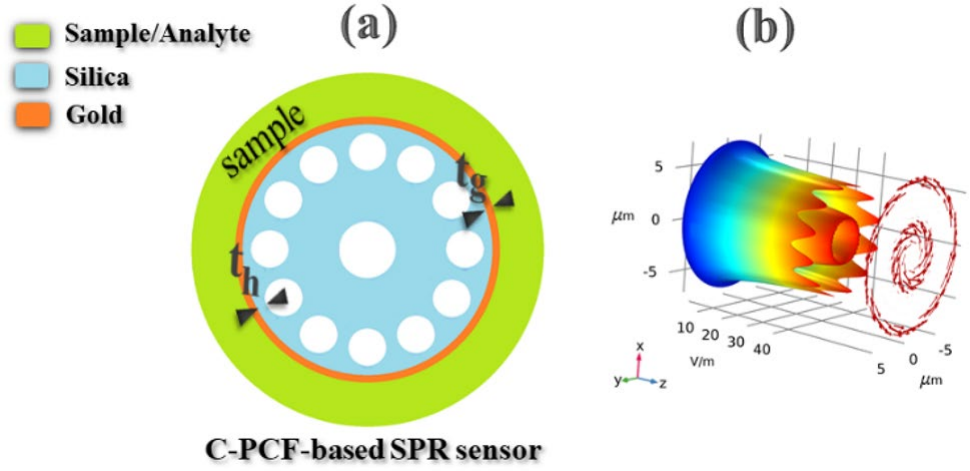
(**a**) The cross-sectional view of the proposed C-PCF-based SPR sensor; and (**b**) the intensity profile indicating strong coupling between the TE_01_ and the SPP modes. The background material is made of silica (blue region), the white circles are air holes, the red ring is gold coated on the fiber cladding with a thickness of t_g_ and distance of t_h_ to the air holes, and the green region is the sample (analyte).

In this section, we propose a new SPR sensor design based on C-PCFs and provide the related theoretical framework. As shown in Fig. 1a, the proposed C-PCF-based SPR sensor is configured by one ring of cladding air holes where the position of each hole with a diameter of *d* = 1.6 µm in the polar coordinate ($R\text{,}\varphi$) is defined by *R* = 4 µm and $\varphi =n\pi /6$ (*n* = 1, 2, …, 12). The center air hole (white circle) with a diameter of *D* = 2.4 µm is located to form the ring core with a thickness of 2 µm. Silica (blue region) is chosen as the background material where its refractive index is well defined by the Sellmeier equation (i.e., 1.444 at 1550 nm)^30^. The mentioned parameters in designing C-PCF configuration are optimized so that the best quality of the TE_01_ mode is propagated in the core. Finally, the proposed sensor is coated with a thin layer of gold (red ring) with a thickness of *t*_*g*_ where the distance to the air holes is *t*_*h*_. By considering a reasonable range of *t*_*g*_ and *t*_*h*_ in the simulations, the most optimal parameters for sensing red/white blood cells are obtained 44 nm and 0.350 µm, respectively. The ring core of the sensor tends to generate and guide two cylindrical vector modes of TM_01_ and TE_01_. As shown in Fig. 1b the TE_01_ mode exhibits a cylindrical intensity profile with an azimuthal polarization which is able to cover the plasmonic surface around the core. Therefore, the evanescent field of the TE_01_ mode well excites the metallic surface and generates the SPP mode and as a result, strong coupling between the TE_01_ and the SPP mode takes place.

**Figure 2:**
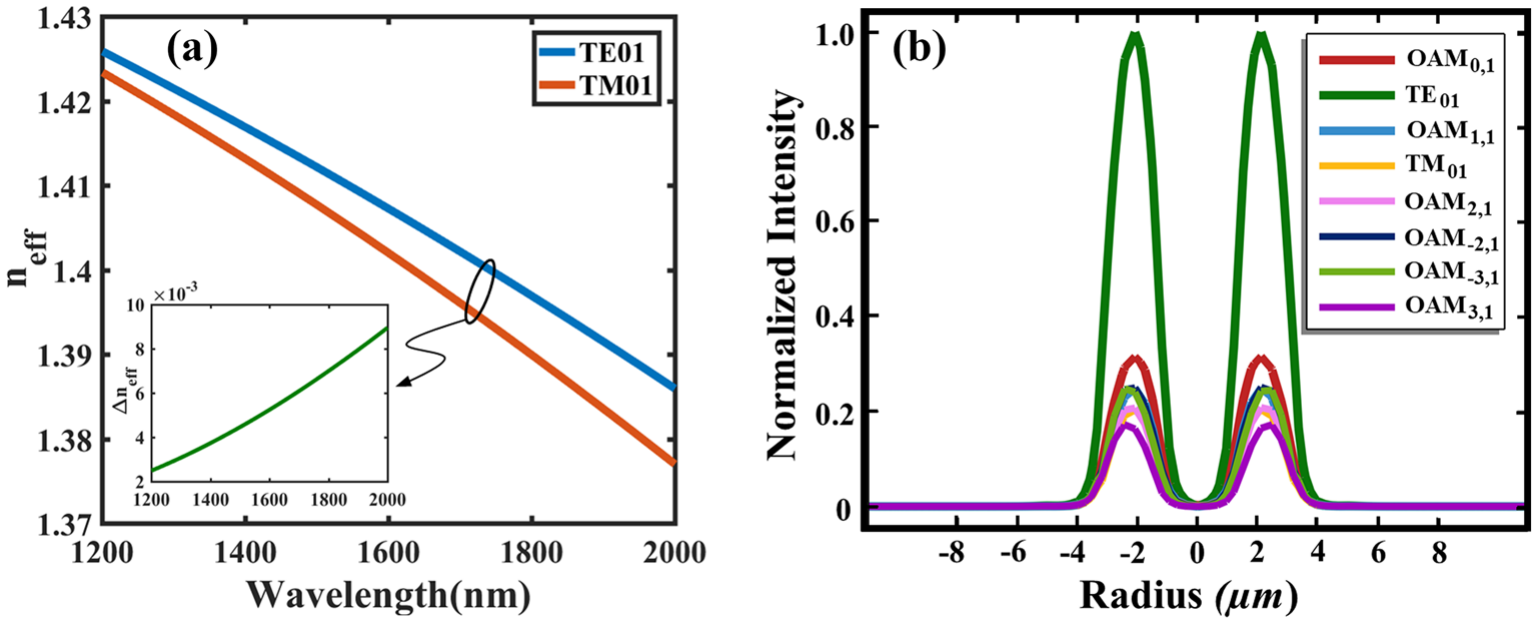
(**a**) The effective index of TE_01_ and TM_01_ modes; the inset shows the effective index difference ${\Delta n}_{eff}$ between two cylindrical modes which is dramatically larger than ${10}^{-4}$; and (**b**) normalized intensity of TE_01_, TM_01_ and OAM_*l,m*_ modes while the same input intensity is assumed.

The cylindrical vector modes (TE_01_ and TM_01_) are the only group (even and odd) that can propagate independently in the C-PCFs while the other higher-order modes groups (even and odd) are degenerate and each group can generate OAM modes. In fact, the difference of mode propagation constant ($\Delta \beta ={\Delta n}_{eff}\omega /c$) between the TM_01_ and the TE_01_ at the angular frequency of $\omega$ is large enough to propagate individually. This claim is proved in Fig. 2a such that the effective index difference ${\Delta n}_{eff}$ between two cylindrical modes is dramatically larger than ${10}^{-4}$ which is the threshold of coupling degenerate modes according to experimental results^33^. This is while in similar SPR sensors, either one even or odd mode (*x* or *y*-polarized) is used which is considered as a drawback because of mode coupling degeneracy. Another advantage of azimuthally polarized mode is that the TE_01_ mode contains the major part of input light intensity. Figure 2b shows that with the same input light intensity, the normalized intensity of TE_01_ is at least three times larger than that of TM_01_ and OAM_*l,m*_ modes; where integer *l* denotes topological charge and $m\ge 1$ shows radial order of intensity profile^4^. This nature of TE_01_ is useful for easier detection in spectrometers or optical spectrum analyzers (OSA).

**Figure 3:**
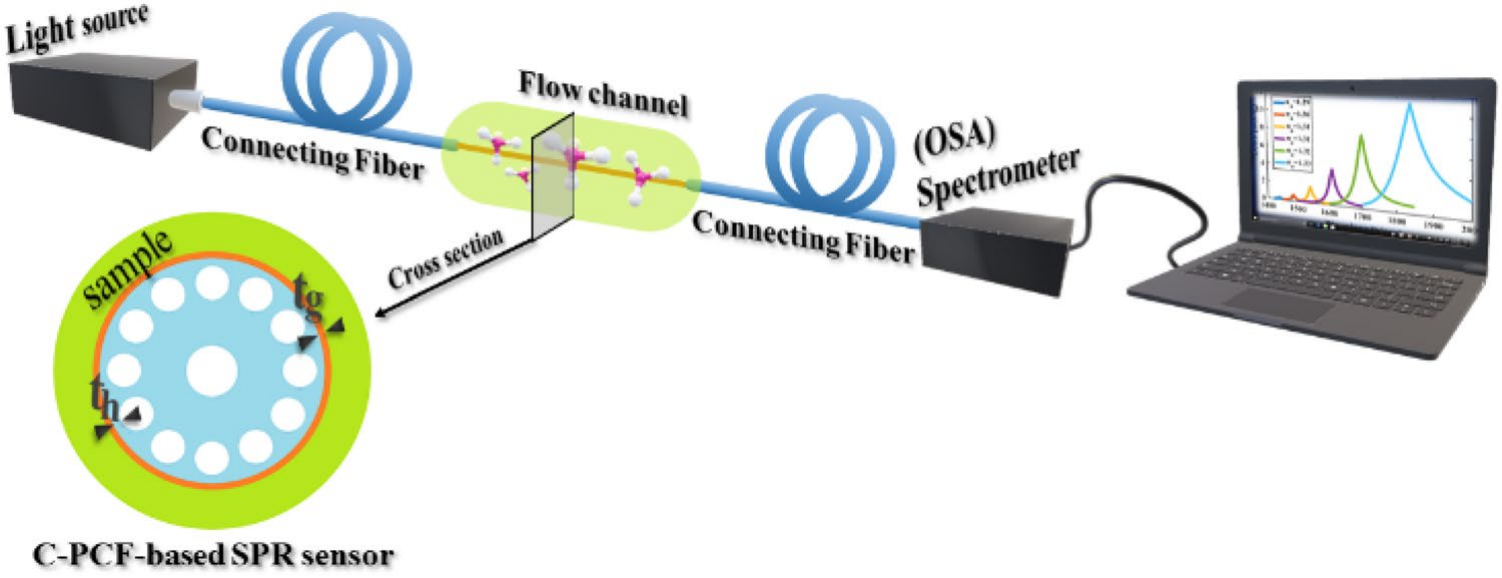
The schematic illustration of the proposed sensor in an experimental setup.

**Figure 4:**
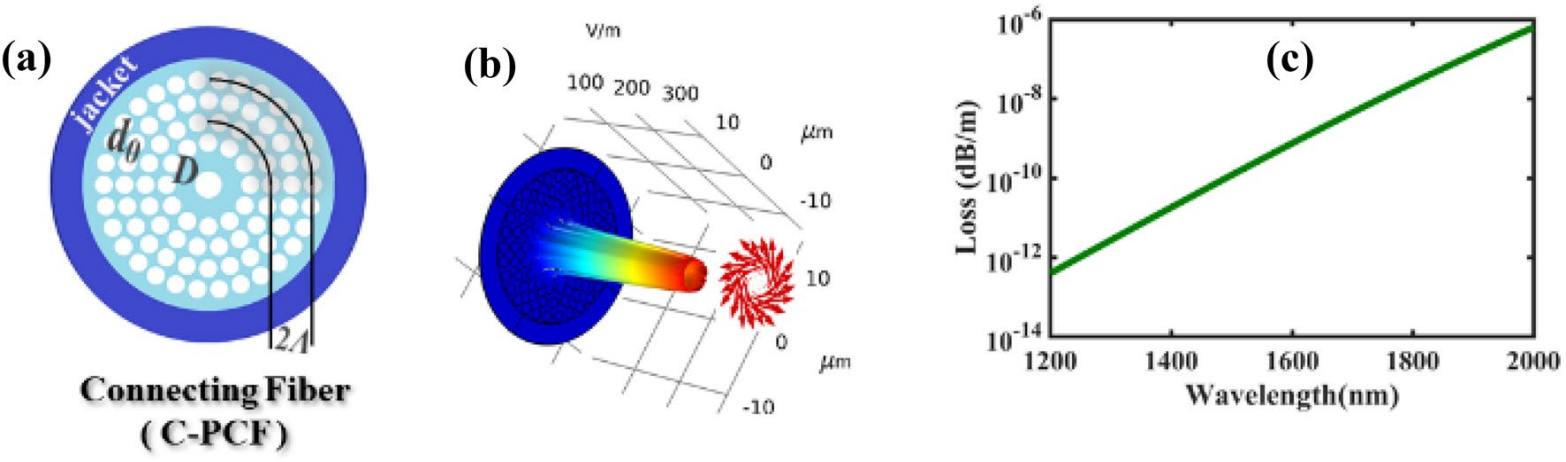
(**a**) The cross-section of the connecting C-PCF with the center air hole diameter of *D* = 2.4 µm, cladding air hole diameter of *d* = 1.6 µm and the pitch size of *Λ* = 2 µm; (**b**) the cylindrical intensity profile of the TE_01_ mode; and (**c**) very low confinement loss spectrum of the TE_01_ mode.

Although this work is a theoretical study, we also propose the schematic experimental setup shown in Fig. 3 to justify our simulation results. By putting the sensor in a flow channel of the sample (analyte), all the transmission properties of the TE_01_ mode undergo red/blue shifts when the sample characteristics are changed. The modified TE_01_ mode can be transmitted to the OSA/spectrometer using the connecting C-PCF shown in Fig. 4a. In fact, the connecting fiber which is the same as one that we have already studied in Ref.^4^, could be different from the sensing fiber (Fig. 1) only in the cladding region. As shown in Fig. 4a, four concentric rings of air holes with a pitch size of *Λ* = 2 µm are placed in the cladding region of the connecting fiber.

The cylindrical intensity profile of the TE_01_ mode is depicted in Fig. 4b while its very low confinement loss (${10}^{-13}dB/m$ to ${10}^{-6}dB/m$) is shown in Fig. 4c over the wavelength range of 1200–2000 nm. The outstanding advantage of the C-PCF-based SPR sensors is that the sensor core is the same as the connecting fiber core to avoid coupling loss. Practically, we hope that the new generation of C-PCF based SPR sensors can meet the requirements using mentioned characteristics of the TE_01_ mode.

One of the best methods to simulate photonics devices is the finite-element method (FEM) which has high accuracy in solving Maxwell’s equations even for complicated structures^12^. The merit of the FEM is that the mesh configuration is formed as triangular elements where by normalizing the size of mesh elements all borders of structure can be identified in nodes well. In this research, all simulations have been done based on the FEM in which a circular perfectly matched layer (PML) with a thickness of 1 μm completely covers the outer surface of the sensor in simulations. Although PML is not a boundary condition, it is a domain that absorbs all outgoing waves (propagating waves and evanescent fields) and can well satisfy absorbing boundary conditions (ABCs). On the other hand, the PMLs are theoretically non-reflecting. The mesh structure is extremely fine such that the maximum element size on uniform domains (namely PML) is 144 nm and the minimum element size on critical domains (namely gold thickness) is 0.288 nm. Drude-Lorentz model is used to evaluate the dielectric constant of gold covering as^14^:

1 $$\varepsilon_{Au} = \varepsilon_{\infty } - \frac{{\omega_{D}^{2} }}{{\omega \left( {\omega + i\gamma_{D} } \right)}} - \frac{{\Delta \varepsilon \Omega_{L}^{2} }}{{\left( {\omega^{2} - \Omega_{L}^{2} } \right) + i\Gamma_{L} \omega }}$$

where the first term shows high-frequency permittivity with *ε*_*∞*_ = 5.9673, the second term has two constant parameters as plasma frequency of *ω*_*D*_ = 13,280.14 *THz* and damping frequency of *γ*_*D*_ = 100.02 *THz*. The last term consists of three constant parameters with names as weighting factor of *∆ε* = 1.09, oscillator strength of *Ω*_*L*_ = 4084.51 *THz* and spectral width of *Γ*_*L*_ = 658.85 *THz*. The simulation results based on FEM cover the complex effective index of modes over the wavelength range of 1200–2000 nm; whereas mentioned the connecting fiber and sensing fiber can support modes with satisfactory optical properties. The loss spectrum of modes in units of *dB/m* can be calculated by the imaginary part of the effective index as^3^:

2 $$\alpha \left( \frac{dB}{m} \right) = 8.686 \frac{\omega }{c}Im\left( {n_{eff} } \right) \times 10^{6}$$

The thickness of gold coating *t*_*g*_ and the distance of *t*_*h*_ to the air holes in Fig. 1a are two important design parameters that can be adjusted according to the sensing range to access the high sensitivity of the sensor. The impact of both *t*_*g*_ and *t*_*h*_ will be investigated in the next section.

Reviewing fabrication methods of PCFs and fiber-based SPR sensors indicates that the proposed sensor can be formed in two steps with fewer challenges than other fiber sensors. The first step is the fiber fabrication process which is done by the stack-and-draw technique^34^. In this process, a preform of the desired configuration with a diameter of 2–3 mm is located in a drawing tower. The high temperature of the furnace (${\sim 2100}^{^\circ }C$) installed in the drawing tower melts the glass and the gravity in the tower leads to a glass drop that forms the desired C-PCF. The preform can only be achieved by stacking a ring of capillaries, and this simplicity in configuration can increase the accuracy of the fabrication as well as fiber stability and reduces the challenges during the fabrication process. The second step is the gold deposition on the outer surface of the C-PCF which can be applied by a magnetron sputtering device^25,26,35^. However, the majority of reported SPR sensors, especially the D-shaped SPR sensors, require an additional step in the fabrication process to achieve the side polish which makes it more complicated and reduces the stability of the sensor. The Comparison between simulation and experimental results of fabricated D-shaped SPR sensors in Refs.^25,26,35^ indicates that FEM simulation predicts the experimental results with high accuracy. Regarding this approach, we expect that our FEM simulation results are well-matched with experimental data.

## Simulation results and discussion

**Figure 5:**
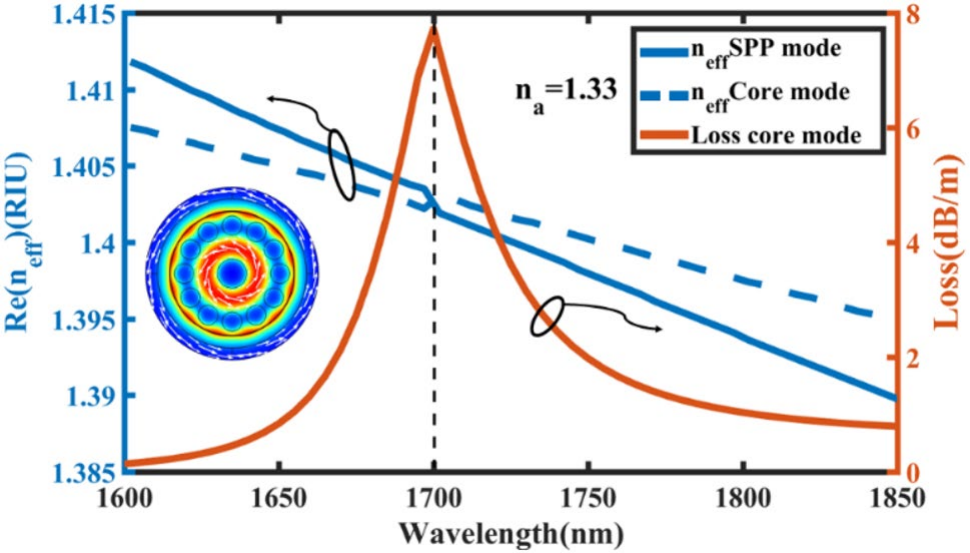
The real part of the effective index for the cylindrical vector mode of the core (dashed blue line), SPP mode (solid blue line) and the loss spectrum of core mode (solid red line) for the C-PCF-based SPR sensor with *t*_*g*_ = 44 nm, *t*_*h*_ = 0.35 µm. The inset shows mode intensity with white arrows indicating electric field at 1700 nm where the complete mode coupling happens.

**Figure 6:**
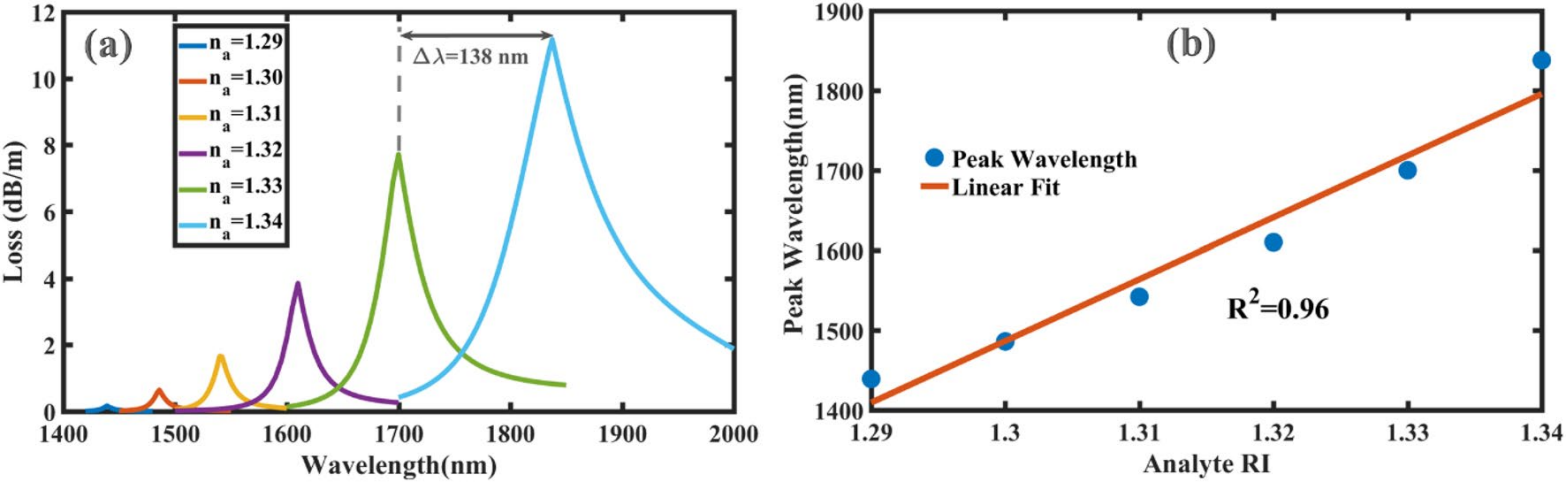
(**a**) Loss spectra for various analyte RI (*n*_*a*_) ranging from 1.29 to 1.34 by step size of 0.01; and (**b**) linear fit (red line) for peak wavelengths (blue points) versus analyte RI.

The proposed SPR sensor is simulated for all logical geometrical values from 40 to 60 nm for *t*_*g*_ and 0.1 µm to 0.4 µm for *t*_*h*_. Although any selection of both ranges shows the sensitivity on the wavelength range from 1200 to 2200 nm, the most sensitivity appears for *t*_*g*_ = 44 nm, *t*_*h*_ = 0.35 µm. In fact, any possible tolerance in *t*_*g*_ and *t*_*h*_ during the fabrication process does not affect the performance of the sensor in general. Obviously, the complete coupling between core and SPP modes occurs when the effective indices (*n*_*eff*_) of modes are equal to each other and as a result two modes change completely to one mode as shown in the inset of Fig. 5. This figure reports the sensor with analyte RI of *n*_*a*_ = 1.33 such that the effective indices of SPP and core modes getting approach to each other in the wavelength range of 1600 nm to 1700 nm and then meet at 1700 nm. This is why the loss spectrum of the core mode (TE_01_) with values on the right vertical axis of Fig. 5 exhibits a sharp peak exactly at 1700 nm indicating the strong field coupling between SPP and core modes such that the whole surface of the gold layer can affect the coupling performance. Therefore, the resonance of the gold layer can be affected by putting the sensor in the flow channel where the sensor experiences different loss spectra depending on different analyte materials in the flow channel (see Fig. 6).

The proposed sensor can operate in a refractive index environment from *n*_*a*_ = 1.29 to 1.34 which is an advantage since this range has received less attention so far. It is obvious in Fig. 6a that by increasing the refractive index of the analyte with a step size of 0.01, the peak wavelengths experience red shift indicating the shift of phase matching condition to higher wavelengths. The most interesting feature of Fig. 6a is that all peaks are well distinguished even for a very small RI change where the maximum resonance wavelength shift is ${\Delta \lambda }_{peak}$=138 nm when the analyte RI changes from 1.33 to 1.34. This makes our proposed sensor very suitable for biophotonics applications since the maximum performance of the sensor occurs in biochemical interactions near the RI of water from 1.33 to 1.34 or1.35 ^10,14,36^. Specifically, regarding the sensing range, the proposed sensor is expected to show high sensitivity for the concentration of red and white blood cells, including human deoxygenated/oxygenated hemoglobin^37–39^, lymphocytes and monocytes^40^ which are effective in diagnosing the progress of cancer tumors^41^. Also, the proposed sensor can provide high efficiency for sensing different liquids such as heavy water D_2_O^42^ and the measuring concentration of heavy metal ions (Hg^2+^, Cu^2+^, Pb^2+^ and Zn^2+^) in water^43^. The increase of loss with increasing analyte RI is logical because the sensor experiences a lower difference between the refractive index of the core and SPR modes so that the maximum loss of 11.18 dB/m occurs at 1838 nm for *n*_*a*_ = 1.34. In addition, our proposed sensor shows very low values of loss peaks comparing other sensors and this merit leads to confining power in the core to be transferred to the connecting fiber and finally detected by OSA.

To investigate other performance characteristics of the proposed sensor, wavelength interrogation (WI) is calculated as a measure to determine the sensitivity of SPR sensors defined as^10^:

3 $$S_{\lambda } \left[ {\frac{nm}{{RIU}}} \right] = \frac{{\Delta \lambda_{peak} }}{{\Delta n_{a} }}$$

where ${\Delta \lambda }_{peak}$ is the resonance peak shift over the variation of the analyte RI: ${\Delta \mathrm{n}}_{\mathrm{a}}$. Using Eq. (3) for the results of Fig. 6a, the proposed sensor indicates the sensitivities of 4700, 5600, 6800, and 9000 nm/RIU at analyte RI of 1.29, 1.30, 1.31, 1.32 respectively, and the maximum sensitivity of 13,800 nm/RIU in the sensing range of 1.33–1.34. The peaks wavelengths are depicted in Fig. 6b with blue dots together with the linear fit (red line). The linearity is very good as *R*^*2*^ = 0.96 with the following linear equation:

4 $$\lambda_{peak} = 7728 \times n_{a} - 8560$$

which shows a high linear sensing response. Theoretically, to calculate the resolution (*R*) of the proposed sensor, the following equation is used^19^:

5 $$R\left[ {RIU} \right] = \Delta n_{a} \frac{{\Delta \lambda_{min} }}{{\Delta \lambda_{peak} }}$$

where the minimum value of spectral resolution of $\Delta {\lambda }_{min}=0.1nm$ and analyte RI variation of $\Delta {n}_{a}=0.01$ are assumed.

The maximum resolution is $R=7.24\times {10}^{-6}$ when the maximum peak shift of ${\Delta \lambda }_{peak}=138nm$ is used (see Fig. 6a). This high resolution means that a very small change of analyte RI in the order of ${10}^{-6}$ can be detected which is comparable to that of other high-resolution sensors reported in Refs.^10,14^. Another measure for the sensor performance is amplitude interrogation (AI) which is more convenient than the wavelength interrogation (WI) method since the AI method does not require spectral analysis and performs in a specific wavelength^32^. Hence, in practice, the challenge of supercontinuum generation of light sources can be reduced in the AI method compared to WI^44^. The amplitude sensitivity is calculated as the loss difference of two spectra, $\partial \alpha (\lambda \text{,}{n}_{a})$, due to two adjacent analyte RIs, $\partial {n}_{a}$, divided by loss spectra, $\alpha (\lambda \text{,}{n}_{a})$, as follows^10^:

6 $${\text{S}}_{{\text{A}}} \left[ {\frac{1}{{{\text{RIU}}}}} \right] = - \frac{{\partial \alpha \left( {\lambda ,{\text{n}}_{{\text{a}}} } \right)}}{{\partial {\text{n}}_{{\text{a}}} }} \frac{1}{{\alpha \left( {\lambda ,{\text{n}}_{{\text{a}}} } \right)}}$$

**Figure 7:**
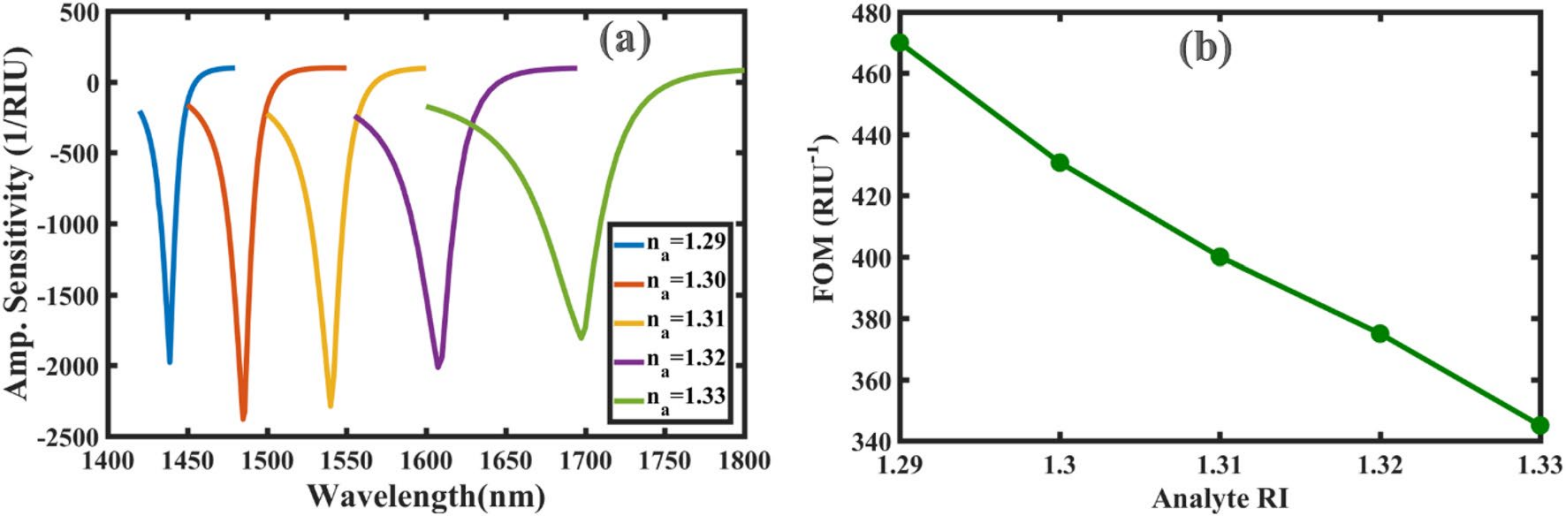
(**a**) Amplitude sensitivity of the proposed SPR sensor for different analyte RIs; and (**b**) the calculated values of FOM for the analyte RIs from 1.29 to 1.33.

From Eq. (6), it is concluded that ${S}_{A}$ increases when the loss spectra are more pronounced. Fortunately, as shown in Fig. 6a, the proposed sensor exhibits very distinct and sharp peaks with changing the analyte RI. The amplitude sensitivity of the proposed sensor is shown in Fig. 7a for different analyte RIs. Maximum amplitude sensitivity is calculated equal to 2380 RIU^−1^ for the analyte RI of 1.30 at 1485 nm; while for the other analyte RIs of *n*_*a*_ = 1.29, 1.31, 1.32 and 1.33, the maximum amplitude sensitivities are 1978, 2287, 2014 and 1807 RIU^−1^ at the wavelengths of 1439, 1540, 1608 and 1698 nm*,* respectively. To the best of our knowledge, C-PCF based SPR sensor illustrates the highest amplitude sensitivity among the reported SPR sensors in literature. Also, the resolution of the sensor can be achieved as $1.11\times {10}^{-5}$ RIU by assuming the detection of 1% for the minimum change of transmitted intensity. Performance characteristics of the proposed SPR sensor together with comparison to other highly sensitive sensors are listed in Table1. As it is seen, besides extremely high amplitude sensitivity, the rest of sensing parameters such as the wavelength sensitivity and the resolution are comparable to other high-sensitivity sensors reported in Refs.^10,14,30^.

**Table 1:** Performance comparison of different sensors reported as highly sensitive SPR sensors.

Ref	Sensor structure	RI range	Amplitude sensitivity (RIU^−1^)	Wavelength sensitivity (*nm*/RIU)	Resolution
^9^	Hollow core D-shaped fiber	1.33–1.34	120	2900	N/A
^10^	MCFF-based SPR sensor	1.46–1.485	820	23,000	$4.35\times {10}^{-6}$
^14^	Proposed D-shaped sensor	1.33–1.43	1086	46,000	$2.2\times {10}^{-6}$
^16^	Graphene based D-shaped sensor	1.33–1.37	216	3700	$2.7\times {10}^{-5}$
^18^	Silver-graphene based PCF	1.46–1.49	418	3000	$3.33\times {10}^{-5}$
^30^	D-shaped SPR sensor	1.35–1.40	300	17,600	$5.55\times {10}^{-6}$
^45^	ITO based D-shaped sensor	1.33–1.38	74	17,000	$5.8\times {10}^{-6}$
^28^	Graphene-based PCF sensor	1.33–1.37	120	N/A	N/A
This work	SPR C-PCF based sensor	1.29–1.34	2380	13,800	$7.24\times {10}^{-6}$

We have also investigated the figure of merit (FOM) as another feature of the sensor which is defined by the ratio between ${S}_{\lambda }$ and the loss spectra full-width at half-maximum (FWHM), as follows^17^:

7 $$FOM \left[ {RIU^{ - 1} } \right] = \frac{{S_{\lambda } }}{FWHM}$$

The calculated values of FOM are shown in Fig. 7b for the analyte RIs of 1.29- 1.33. The maximum FOM of the C-PCF-based SPR sensor is obtained as 470 RIU^−1^ at *n*_*a*_ = 1.29 while for the rest of the analyte RIs of 1.30, 1.31, 1.32 and 1.33, FOMs are 430.8, 400, 375 and 345 RIU^−1^, respectively. Although few papers have reported the FOM for their proposed SPR sensors, the mentioned value of our FOM is even higher than the maximum FOM values reported in Refs.^17,30,46^.

**Figure 8:**
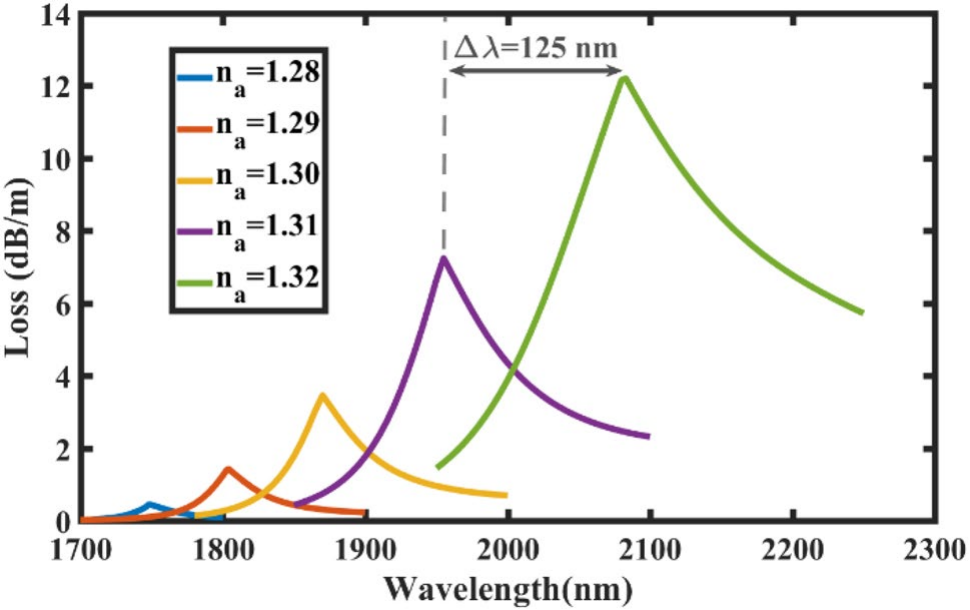
Loss spectra of the proposed SPR sensor simulated at different analyte RIs. The gold thickness is *t*_*g*_ = 60 nm and distance to the air holes is *t*_*h*_ = 200 nm.

As mentioned in Sect. 2, all efforts have been made to keep the core of the sensing fiber the same as that of the connecting fiber in order to alleviate the challenges of coupling mechanism. Hence, we kept the design parameters of the core structure constant to guide the main mode (TE_01_) with high quality. The other two design parameters, *t*_*g*_ and *t*_*h*_, can significantly affect the performance of the sensor, where we have selected the most optimal values to achieve the highest sensitivity. Nevertheless, the simulation results for a different set of *t*_*g*_ = 60 nm and *t*_*h*_ = 200 nm are shown in Fig. 8 where the analyte RIs range is 1.28–1.32 (instead of 1.29–1.34) and the wavelength range is 1700–2200 nm (instead of 1400–2000 nm as for the optimal parameters). As it is evident, the maximum wavelength sensitivity is reduced from 13,800 to 12,500 nm/RIU and FWHM of the loss spectra are increased which can decrease the amplitude sensitivity and resolution of the sensor as well. Although we have prioritized achieving the maximum performance of the sensor to sense low concentrations of red/white blood cells, it is expected that the proposed sensor will cover a wider range of wavelengths and RI of the analyte. To avoid this limitation and meet the needs of other ranges of sensitivity and wavelength, we expect that this generation of SPR sensors can be further developed by utilizing emerging fibers such as C-PCFs^2,4,47^ and ring-core fibers^48^. Also, it anticipated that the reconfiguration of hollow-core fibers^3^ via adding metallic layers leads to new SPR sensors based on the TE_01_ mode in the terahertz region.

## Discussion

We have proposed a novel structure of the SPR sensor based on C-PCFs in which for the first time, cylindrical vector modes with azimuthal polarization are used. A C-PCF was designed with a ring of air holes in the cladding region coated by a gold layer to excite the SPP mode. The proposed sensor with optimal geometrical parameters was simulated using the FEM method and exhibited strong coupling between the TE_01_ and SPP modes for the analyte refractive indices ranging from *n*_*a*_ = 1.29 to *n*_*a*_ = 1.34. The maximum sensitivity of 13,800 nm/RIU was reported in the sensing range of 1.33–1.34 (that is very close to the RI of water) which is of great interest for biochemical applications. In particular, human blood can be used as the analyte to measure the concentration of hemoglobin, lymphocytes and monocytes, which are crucial in medical diagnosis. A very high resolution showed that the proposed SPR sensor is able to detect the variation of analyte RI in the order of ${10}^{-6}$. Also, amplitude sensitivity and FOM were calculated with maximum values of 2380 RIU^−1^ and 470 RIU^−1^, respectively. The obtained values for the amplitude sensitivity and the FOM are the highest ever reported compared with those of other SPR sensors. Also, our sensor simultaneously exhibits high wavelength sensitivity and resolution which are comparable to those of highly sensitive sensors. In general, our high-performance SPR sensor based on cylindrical vector modes in C-PCFs holds great promise for applications in various areas such as biophotonics.

## Data Availability

Data are available from the corresponding author on reasonable request.
